# The Inhibitory Mechanism in Learning Ambiguous Words in a Second Language

**DOI:** 10.3389/fpsyg.2017.00636

**Published:** 2017-04-27

**Authors:** Yao Lu, Junjie Wu, Susan Dunlap, Baoguo Chen

**Affiliations:** ^1^Beijing Key Laboratory of Applied Experimental Psychology, School of Psychology, Beijing Normal UniversityBeijing, China; ^2^State Key Lab of Cognitive Neuroscience and Learning, Beijing Normal UniversityBeijing, China; ^3^Children’s Learning Institute, University of Texas Health Science Center at Houston, HoustonTX, USA

**Keywords:** second language, ambiguous word leaning, inhibitory control, deliberate learning, semantic representation

## Abstract

Ambiguous words are hard to learn, yet little is known about what causes this difficulty. The current study aimed to investigate the relationship between the representations of new and prior meanings of ambiguous words in second language (L2) learning, and to explore the function of inhibitory control on L2 ambiguous word learning at the initial stage of learning. During a 4-day learning phase, Chinese–English bilinguals learned 30 novel English words for 30 min per day using bilingual flashcards. Half of the words to be learned were unambiguous (had one meaning) and half were ambiguous (had two semantically unrelated meanings learned in sequence). Inhibitory control was introduced as a subject variable measured by a Stroop task. The semantic representations established for the studied items were probed using a cross-language semantic relatedness judgment task, in which the learned English words served as the prime, and the targets were either semantically related or unrelated to the prime. Results showed that response latencies for the second meaning of ambiguous words were slower than for the first meaning and for unambiguous words, and that performance on only the second meaning of ambiguous words was predicted by inhibitory control ability. These results suggest that, at the initial stage of L2 ambiguous word learning, the representation of the second meaning is weak, probably interfered with by the representation of the prior learned meaning. Moreover, inhibitory control may modulate learning of the new meanings, such that individuals with better inhibitory control may more effectively suppress interference from the first meaning, and thus learn the new meaning more quickly.

## Introduction

Vocabulary proficiency is an important aspect of second language (L2) ability, and vocabulary learning is crucial to developing L2 proficiency. For learners of a second language, as their proficiency improves, they are constantly encountering and learning new words or new meanings of already known words. Therefore, it is important to understand the processes involved in L2 vocabulary acquisition. In the present study, we focus on meaning relations of L2 ambiguous words during learning.

In the literature on word learning, most previous studies focused on the learning of monosemous words, for which one form is mapped to one meaning. However, in English, most words have multiple meanings. Additionally, current words continue to acquire new meanings, for example, ‘tweet’ and ‘tablet’ have recently acquired novel technological and social-media meanings. Moreover, when a word with multiple meanings is translated into another language, the multiple translations in that language would possibly belong to different lexicons, creating mismatches. A major barrier to L2 vocabulary learning is the mismatches between L1 and L2 at the lexical level (MacWhinney, 1997; Tokowicz et al., 2002; Prior et al., 2007; Tokowicz, 2014). For example, the English word “sentence” refers to both “a group of words which, when they are written down, begin with a capital letter and end with a period, question mark, or exclamation mark” and “the punishment that a person receives after they have been found guilty of a crime.” Unsurprisingly, for a Chinese learner of English, he will find no corresponding word in Chinese that express the exact two meanings of “sentence,” instead, two words, “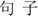 (juzi)” (the grammatical meaning) and “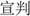 (xuan pan)” (the legal meaning) to represent the two meanings of sentence, separately.

There are different kinds of semantic ambiguity in traditional linguistic research, such as homonym or polysemy. However, the essence of one-to-many mappings is that a single word form is matched to several meanings. For example, the polysemous word ‘run’ has many different dictionary definitions. The multitude of ways in which we use these words to express a range of subtly different concepts is developed through general principle of meaning extension by extending its meaning to a similar situation and plausible reasoning, and the semantic distance among multiple meanings could range from close to far (Klein and Murphy, 2001). In fact, recent psycholinguistic studies (Klein and Murphy, 2001; Klepousniotou et al., 2008; Jager et al., 2016) have suggested that ambiguity could be measured continuously, i.e., the relatedness between meanings is a continuum rather than a dichotomy, with polysemy falling between the two ends of meaning relatedness: homonym (least related) and synonym (most related). Therefore, the term “ambiguous words” is employed as a joint concept referring to both polysemy and homonyms. Here in the current study, L2 ambiguous words with two unrelated meanings were used as study items.

Children have long been shown to have problems in learning the multiple meanings of polysemous words (Slobin, 1985; Merriman et al., 1989; Mazzocco, 1997; Woodward and Markman, 1998; Doherty, 2004). For example, Doherty (2004) investigated children’s ability to learn a new meaning for a known word, i.e., a pseudo-homonym. The word “fork,” for instance, was introduced by a story as referring to a different object. Children were asked to select the intended referent according to the label. Results showed lower accuracy when a pseudo-homonym served as the referent’s label compared to a non-word. Children therefore have difficulty in associating a new meaning to a word they already know. The author suggested that the children were poor at learning new meanings for pseudo-homonyms because (1) they prefer unique mappings between form and meaning, and (2) they suffer from poor executive control, and thus they had difficulty suppressing the primary meaning of the homonyms.

This one-to-many mapping may continue to pose difficulty for adults learning a second language. Adult second language learners are faced with a particular challenge in that adults are influenced by the transfer of information from an ingrained L1 system to newly learned L2 (MacWhinney, 1997; Tokowicz and MacWhinney, 2005; Tokowicz, 2014). For example, an adult Chinese learner of English, who already has a fully formed and deep-seated lexical and semantic system, will have to integrate “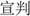 (xuan pan) (the legal meaning of sentence)” with “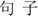 (juzi) (the grammatical meaning), which are distantly related to each other either in lexical representation or in semantic space of his native language, to a single form “sentence” in English. Revising the existing lexical and semantic system is needed to accommodate the new word.

However, to our knowledge, there are no such studies focusing on relations of the multiple semantic representations of L2 ambiguous words, and thus little is known about what mechanism may be involved in L2 ambiguous word learning. One possible reason underlying the difficulty is the order of acquisition, i.e., the new meaning has to compete with entrenched one-to-one mappings. A study using L1 ambiguous words provides insights for the difficulty of ambiguous word learning (Degani et al., 2014). In a multiple-session training study, native English speakers learned foreign Dutch vocabulary items that mapped to English either in a one-to-one way, such that one Dutch word corresponded to one English translation, or in a one-to-many way, such that two Dutch words corresponded to a single English translation. Critically, for the ambiguous words, these two Dutch words were taught on consecutive trials. Results showed that ambiguous words were produced and recognized substantially less accurately than unambiguous words, and that the translation learned first enjoyed a considerable advantage over the translation learned second.

Nonetheless, a point that needs to be addressed is that the ambiguous words used in Degani’s study belong to L1, in which case both meanings of the English word were most likely already distinguished for native English-speaking participants (e.g., “change,” the monetary meaning and the alteration meaning). Therefore, when learning to map a Dutch translation to each of these meanings, the learner can avoid the one-to-many mapping problem, and in essence, establish one-to-one mapping (Degani and Tokowicz, 2010). Therefore, these results cannot account for the mechanism when a new meaning is integrated into a one-to-one mapping (i.e., a monosemous word), the focus of the present study. In contrast, the ambiguous words adopted in the current study belong to L2, in which case two separately represented meanings, which have already been established in semantic space, need to converge to one single form in L2. In short, semantic representations need revising in the learning of L2 ambiguous words (the present study), but not in the learning of L2 words that correspond to each meaning of L1 ambiguous words (as in Degani’s study).

Overall, the difficulty of learning the new meaning of an ambiguous word seems to be rooted in the process of integrating the new meaning into the pre-established one-to-one form-meaning mapping, such that the stable representation of the primary meaning may interfere with the establishment of the newly come competitor. However, so far there is no direct evidence for this inhibitory connection.

As for how to build up vocabulary, there are several ways to acquire lexical knowledge. One common way of building up L2 vocabularies is deliberate learning, i.e., direct mapping between a novel word and a meaning (or a translation equivalent in L2 word learning). Deliberate learning is often embodied in the form of flashcard learning, which involves repeated retrieval of the form and meaning of a word (Oxford and Crookall, 1990). In a typical flashcard learning study (Perfetti et al., 2005), college students were required to study 60 rare English words for 45 min with the presentation of the word on one side of the card and a brief definition on the other side. Following the study phase, participants made meaning judgments on pairs of words while event-related potentials (ERPs) were recorded. The prime word was a trained rare word, an untrained rare word, or a familiar word, and the target word was a semantically related or unrelated word. For trained words and familiar words, results showed faster responses for related words compared to unrelated words, and that the related words elicited a smaller N400 than the unrelated words. Note that in psycholinguistic literature, the amplitude of N400 is an index of the ease in integrating the semantic information into context (Kutas and Hillyard, 1980; Holcomb and Neville, 1990; Nobre and McCarthy, 1994; Rolke et al., 2001; Lau et al., 2008; Kutas and Federmeier, 2011). The semantic relatedness effect reflected in faster responses and in N400 effects suggests meaning could be obtained through deliberate learning, and semantic representation could be established by directly mapping a meaning with a novel word form.

One study using L2 flashcard learning demonstrated robust establishment of L2 lexical representations (Elgort, 2011). In this study, participants were advanced learners of English whose native languages were not controlled for. Participants were asked to learn the meaning and the pronunciation of 48 novel English words during the initial learning session. Then they were given a set of word cards to take home to practice form-to-meaning and meaning-to-form retrieval following a suggested spaced-repetition schedule for 1 week. Newly learned English pseudowords were used as form primes and semantic primes in three lexical decision tasks, and form and semantic representations were probed based on their ability to generate form and semantic priming effects. The results showed that the formal-lexical and lexical semantic representations of deliberately studied L2 words were established, and could be retrieved fluently in a lexical decision task. Another common way of building up L2 vocabularies is bilingual flashcards, with L1 translation equivalents or definitions, instead of L2 definitions. A follow-up study conducted by the same research team (Elgort and Piasecki, 2014) replicated the training regime and experimental design and further explored the effectiveness of using bilingual flashcards in L2 word learning. In this study, adult German–English bilinguals learned English pseudowords using bilingual flashcards. Again, quality of semantic representations established for the studied items was tested using semantic priming lexical decision task. The analyses also investigated whether the learning outcome was modulated by participants’ L2 lexical proficiency. The results showed that only bilinguals with large L2 vocabularies established semantic representations for novel words. Other studies have also revealed the effectiveness of deliberate learning in establishing lexical representations for novel words (Baxter, 1980; Oxford and Crookall, 1990; Hulstijn, 2003; Duyck and Brysbaert, 2004; Perfetti et al., 2005; Nation, 2007; Elgort and Nation, 2010; Ghazi Saidi et al., 2013).

In summary, the current study aimed to investigate L2 ambiguous word learning from the perspective of how a new meaning is added to lexical representations and what the consequent relations between the prior and the new would be, at the initial stage of lexical learning. In addition, we are further interested in whether individual differences in inhibitory ability modulate the L2 ambiguous word learning outcome. Our question is: If the new meaning competes with the pre-existing meaning, are the newly established connections between the new meaning and the former meaning mutually inhibitory? Or, is the inhibitory connection unidirectional, such that the former meaning would interfere with the integration of the new meaning? And lastly, does inhibitory ability play a beneficial role in L2 ambiguous word learning?

To address the above mentioned questions, we used a deliberate learning method to investigate what the relationship between the representations of the new and the prior meanings would be at the initial stage of L2 ambiguous word learning, as well as examining how inhibitory control functions during L2 ambiguous word learning. The deliberate learning method was embodied in an artificial word learning paradigm using bilingual flashcards. Participants were low-to-intermediate Chinese–English bilinguals. Instead of comparing unambiguous words with ambiguous words in a general manner, we separated the two meanings of ambiguous words and set each meaning as a separate condition. Therefore, three conditions of pseudoword-meaning pairs were created, namely unambiguous words, first meaning of ambiguous words, and second meaning of ambiguous words. The decision to use pseudowords rather than real words was made to ensure that participants were not exposed to the new words under any circumstances or had partial knowledge of the words they were required to learn. All the pseudoword-meaning pairs were required to be learned in four consecutive days. Inhibitory control was introduced as a subject variable measured by a color word Stroop task, to provide direct evidence for the inhibitory connection between the semantic representations of multiple meaning, and consequently to investigate the modulatory role of inhibitory control in L2 ambiguous word learning.

A semantic-relatedness judgment task was used to assess the learning effects of novel words, in which participants read a pair of words and judged whether the two words were semantically related or not (Perfetti et al., 2005; Chen et al., 2014). The reason to use a semantic-relatedness judgment task rather than a lexical decision task is that it is hard for low-to-intermediate bilinguals to establish the representation in an automatic manner as revealed by a semantic priming LDT (Meyer and Schvaneveldt, 1971; McRae et al., 2005). A number of studies have demonstrated that a certain threshold level of L2 proficiency is needed for reliable automatic priming effects to occur, as these effects rely on the participants’ ability to access and process lexical representations in an automatic manner (Kroll and Stewart, 1994; Bijeljac-Babic et al., 1997; Van Hell and Dijkstra, 2002). However, in judging the semantic relationship between the prime and the target, participants are forced to integrate the novel words into the semantic network through the connection with related/unrelated concepts or features. Therefore, for low-to-intermediate learners of the English language, we hold that a semantic relatedness judgment task is more effective at testing the semantic status of the newly learned words.

Our predictions were that (1) the representation of the primary meaning would interfere with the establishing of the new meaning, and thus the semantic representation of the new meaning would be unstable at the very early stage of learning, and (2) inhibitory control plays an important role in the learning of L2 ambiguous words, such that individuals with better inhibitory control ability may more effectively suppress the interference from the primary meaning and thus learn the new meaning more quickly.

## Materials and Methods

### Participants

Prior to data collection, ethical approval was obtained from the Committee of Protection of Subjects at Beijing Normal University. All participants signed the written informed consent form. Participants were 49 right-handed, Chinese native speakers (10 males; mean age 21.75 years) with low to intermediate proficiency in English, ranging in education level from first year of college students to a graduate degree. They were recruited from several universities in Beijing and received payment for their participation. All participants reported having normal or corrected-to-normal vision. Data from a total of five participants were excluded due to technical problems during training (one participant) or low accuracy in the test phase (four participants). Analyses were conducted on the final set of 44 participants.

English proficiency of each participant was assessed by the Oxford Placement Test (OPT) and self-assessment ratings. The Oxford Placement Test includes 25 multiple choice questions and a cloze test, with a maximum score of 50. Self-ratings of English abilities were given on a 6-point scale ranging from 1 (L2 skills are much worse than L1 skills) to 6 (L2 skills are just as good as L1 skills). Participants’ average ratings and OPT scores are shown in **Table 1**.

**Table 1:** Mean (SD) age of acquisition (AoA) of English L2, Oxford Placement Test (OPT) scores, and English L2 self-ratings of participants.

		English level self-rating			
		
AoA	OPT	Listening	Speaking	Reading	Writing
10.59 (1.44)	39.30 (3.62)	3.20 (1.07)	3.02 (0.95)	2.64 (1.01)	2.86 (1.32)


### Materials

#### Novel Words

Participants were required to learn 30 English–Chinese translations (see Appendix A). Additionally, 30 English pseudowords were created using Wuggy^1^, a pseudoword generator particularly geared toward making non-words for psycholinguistic experiments (Keuleers and Brysbaert, 2010). The 30 pseudowords were evenly split into two sets–unambiguous words and ambiguous words–serving as the novel words to be learnt in English. The unambiguous words (hereafter, unA) were coupled with one meaning in Chinese, e.g., sessand-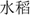 (paddy), and the ambiguous words were coupled with two meanings in Chinese, e.g., soltoor-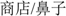 (shop/nose) (hereafter, A-1 stands for the first meaning, and A-2 stands for the second meaning). The two sets of 15 pseudowords were all two-syllable, 6–8 letters in length, and matched in word length and OLD20 (Yarkoni et al., 2008). OLD20 is the average Orthographic Levenshtein Distance between the generated candidate and its 20 most similar words in the lexicon. A smaller value of OLD20 indicates fewer steps (letter additions, deletions, or substitutions) to a real word, such that many words can be made by changing just one or two letters (Keuleers and Brysbaert, 2010). Therefore, pseudowords with low OLD20 values (OLD20 < 2) may introduce a confounding effect of associative memory through a similar word. For example, when learning the pseudoword *peacher*, participants could associate it with an orthographically similar word, such as peach or teacher). Therefore, to reduce associative memory effects while still having pseudowords not too unlike real English words, the OLD20 of the 30 pseudowords in this study was kept at three or higher.

We strove to keep the two meanings of the ambiguous words as semantically unrelated as possible. The similarity of the two senses of the ambiguous words was rated by 20 additional participants from the same background as the participants, in a scale ranging from 1.00 (unrelated) to 7.00 (related). Finally, candidate pairs with scores below 1.6 were selected as the final 15 pairs of meanings (Mean = 1.24, *SD* = 0.153).

We also wanted to make the three sets of meanings comparable (one for unambiguous words, two for ambiguous words), so that any difference between the stimulus types could be only attributed to the representational status, so lexical properties of the three sets of Chinese meanings should be matched. The meanings were all two-character words randomly chosen from the Modern Chinese Frequency Dictionary (1986). The words belong to one of the following semantic categories: furniture, fruit, body part, electrical appliance, clothing, job, animal, natural scene, vegetable, and sports. Then, the same 20 participants (10 males) judged the familiarity and concreteness in a 7-point scale (with 7 indicating the most familiar/concrete, and 1 indicating the least familiar/concrete). Then several repeated one-way ANOVAs were conducted among the three sets of Chinese meaning on familiarity, concreteness, frequency (*Modern Chinese Frequency Dictionary*, 1986), and stroke number, and no significant differences were observed for any of the lexical properties, for familiarity, *F*(2,28) = 0.084, *p* = 0.919, η^2^ = 0.006, for concreteness, *F*(2,28) = 0.002, *p* = 0.998, η^2^ = 0.000, for frequency, *F*(2,28) = 0.579, *p* = 0.567, η^2^ = 0.040, and for stroke number, *F*(2,28) = 0.283, *p* = 0.755, η^2^ = 0.020 (see **Table 2**).

**Table 2:** Lexical properties of three sets of Chinese meanings, mean (SD).

Word type	Familiarity	Concreteness	Frequency	Stroke Number
unA	6.56 (0.23)	6.10 (0.32)	40.41 (29.02)	15.80 (3.67)
A-1	6.53 (0.24)	6.09 (0.18)	32.11 (20.34)	14.74 (3.41)
A-2	6.53 (0.17)	6.10 (0.44)	42.85 (23.17)	15.80 (5.17)


*unA, unambiguous words; A-1, first meaning of ambiguous words; A-2, second meaning of ambiguous words.*

#### Target Words in the Semantic Relatedness Judgment Task

In the testing phase, a cross-language semantic relatedness judgment task was adopted to test the learning outcomes. Each of the 45 meanings was paired with a semantically related Chinese two-character word (serving as the target word), and a semantically unrelated one, respectively, altogether forming six groups of word pairs, 90 pairs in total (see Appendices B and C). Participants were instructed to judge whether the prime (the English pseudoword) was semantically related to the target (the two-character Chinese word). Note that for an ambiguous word, “related” is the correct response if the target word was semantically related to any of the prime’s two nuances of meaning, whereas “unrelated” would be the answer if the target word was semantically unrelated to either of the prime’s two meanings. Again, lexical properties for the target word (i.e., familiarity and concreteness) were rated by the same 20 participants on a scale ranging from 1.00 (least familiar/concrete) to 7.00 (most familiar/concrete). Lexical properties (i.e., familiarity, concreteness, frequency, and stroke number) were summarized in **Table 3** and matched among the six groups as confirmed by a one-way ANOVA. No significant differences were observed for any of the lexical properties, for familiarity, *F*(5,70) = 0.366, *p* = 0.870, η^2^ = 0.025, for concreteness, *F*(5,70) = 1.084, *p* = 0.377, η^2^ = 0.072, for frequency, *F*(5,70) = 0.573, *p* = 0.721, η^2^ = 0.039, and for stroke number, *F*(5,70) = 0.743, *p* = 0.594, η^2^ = 0.050.

**Table 3:** Lexical properties of six sets of target words, mean (SD).

Target word	Familiarity	Concreteness	Frequency	Stroke Number
unA, R	6.49 (0.33)	6.12 (0.37)	59.44 (89.15)	15.40 (3.91)
unA, unR	6.52 (0.18)	6.21 (0.18)	35.20 (58.34)	15.13 (4.57)
A-1, R	6.56 (0.33)	6.07 (0.39)	41.78 (55.36)	17.00 (5.02)
A-1, unR	6.59 (0.23)	6.17 (0.30)	82.70 (193.36)	15.40 (4.45)
A-2, R	6.48 (0.20)	5.98 (0.38)	77.25 (92.35)	15.47 (5.98)
A-2, unR	6.53 (0.23)	6.14 (0.17)	32.70 (63.65)	13.60 (4.01)


*unA, unambiguous words; A-1, first meaning of ambiguous words; A-2, second meaning of ambiguous words; R, semantically related, unR, semantically unrelated. For example, unA, R refers to target words that were semantically related to unambiguous words.*

Then, the same 20 participants rated the semantic relatedness between the meaning of the prime word and the target word. A one-way ANOVA revealed no differences among unA, A-1, and A-2 related pairs (6.27 ± 0.37, 6.26 ± 0.28, 6.17 ± 0.38), *F*(2,28) = 0.401, *p* = 0.674, η^2^ = 0.028, nor among unA, A-1, and A-2 unrelated pairs (1.26 ± 0.13, 1.27 ± 0.16, 1.31 ± 0.14), *F*(2,28) = 0.605, *p* = 0.553, η^2^ = 0.041. However, for related pairs (6.23 ± 0.35) versus unrelated pairs (1.28 ± 0.14), a paired-sample *t*-test reached significance, *t*(44) = 90.127, *p* < 0.001, Cohen’s *d* = 13.435. Furthermore, several paired-sample *t*-tests between related and unrelated pairs for unA, A-1, and A-2 were conducted, and all reached significance. For unA, *t*(14) = 53.452, *p* < 0.001, Cohen’s *d* = 13.801, for A-1, *t*(14) = 56.796, *p* < 0.001, Cohen’s *d* = 14.665, and for A-2, *t*(14) = 46.247, *p* < 0.001, Cohen’s *d* = 11.941.

### Procedure

**FIGURE 1:**
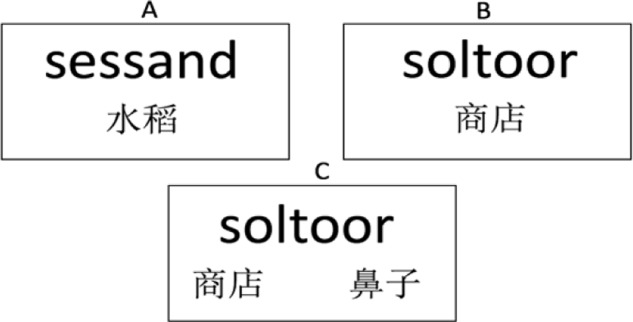
**Illustration of flashcard-like learning of new words.** **(A)** An example of unambiguous word whose learning started from day one. **(B)** An example of ambiguous word and its first meaning, whose learning started from day one. **(C)** An example of ambiguous word with both meanings, of which the second meaning started to be presented from day two.

**FIGURE 2:**
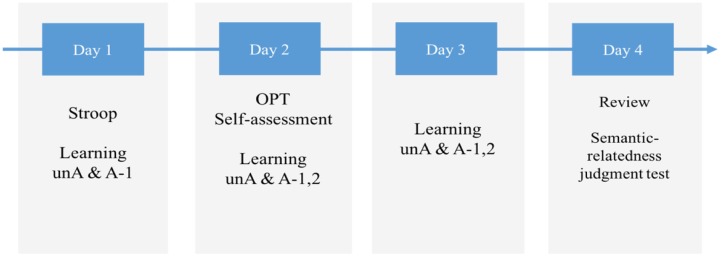
**General procedure for learning.** unA, unambiguous words; A-1, first meaning of ambiguous words; A-2, second meaning of ambiguous words; OPT, Oxford Placement Test.

The experiment took place over four consecutive days, with learning spread across the first 3 days because previous research suggests that sleeping after exposure to new information improves the learning outcomes (Gais et al., 2006; Gómez et al., 2006; Tamminen, 2010). During learning, participants were instructed to look at and memorize the English pseudowords and their Chinese meanings presented on flashcard-like slides on the computer screen (see **Figure 1**). Instructions were as follows: “In today’s learning task, you are going to learn 30 new English words. You can click the slide show and control the learning at your own pace and it will take about 30 min. The English word and corresponding Chinese meaning will appear successively by mouse-clicking. Please follow the instruction, and there will be a simple test after learning.” Learning outcomes were tested by an L2-to-L1 translation production test (e.g., sessand-?, soltoor-?) with paper and pencil on each of the learning days, and by an L2-to-L1 oral translation production test (the experimenter pointed at each pseudoword, and the participant produced the translations orally) on the fourth day. In order to simulate the naturalistic learning of the ambiguous words, the learning task on the first day contained merely the first meaning (A-1) of the ambiguous words, and the study of the second meaning (A-2) began on the second day. Note that on the first day of learning, the 15 ambiguous (A-1) words were effectively unambiguous words. They didn’t become ambiguous for the learners until subsequently learning the second meaning (A-2). The Stroop task took place on the first day before the learning task. The learning outcome measure, i.e., semantic-relatedness judgment task took place on the fourth day. The general procedure of learning and testing is summarized in **Figure 2**.

### Stroop Task

The Stroop task adopted in the present study is adjusted from that used in Qiu et al. (2006) and Heidlmayr et al. (2014). The experiment consisted of neutral, congruent, and incongruent stimuli. The congruent conditions contained the color words (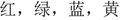) (red, yellow, green, blue) written in the same color with the stimulus meaning [e.g., the word ‘
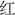
’ (red) written in red color]. The incongruent conditions contained the color words written in a different color not matching the word meaning [e.g., the word ‘
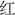
’ (red) written in green ink]. In the neutral condition, three non-color words (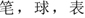) (pen, ball, watch) were displayed in equally varying print colors as in congruent and incongruent conditions. Participants were instructed to rest their left middle, left index on the d, f key and right index and right middle finger on the j, k key on the keyboard, each representing one of the four colors. The procedure was as follows: the fixation point lasted 500–1000 ms (500, 625, 750, 875, 1000 ms, in order to avoid participants’ expectancy of the onset of the stimuli), then the word appeared on the screen until a response was registered or after 1500 ms elapsed with no response, followed by a blank screen for 500 ms. Participants were asked to identify the color in which the stimulus was presented by pressing the button of the corresponding color. The experiment was divided into a practice and a test phase (108 congruent trials, 36 incongruent trials and 36 neutral trials, evenly split into two blocks), presented in a pseudo-randomized order (Nowagk, 1998). Note that no more than three trials of the same experimental condition were presented in succession and no immediate repetition of word or print color. The practice phase was designed to rehearse the mapping of colors onto fingers and the pressing of the response buttons. A short break was granted between two blocks. The “inhibition effect” was defined as the RT difference between the incongruent and the neutral conditions, and smaller inhibition effect indicated better inhibitory control ability (Qiu et al., 2006; Badzakova-Trajkov et al., 2009; Coderre et al., 2011).

### Learning Outcome Measure: Semantic-Relatedness Judgment Task

A cross-language semantic relatedness judgment task was adopted to test the learning outcomes of the novel words. Participants were instructed to judge whether the prime (the English pseudoword) was semantically related to the target (a Chinese word). Note that for ambiguous word, participants were required to press “F” (related) if the target word was semantically related to any of the prime’s two nuances of meaning, whereas “J” (unrelated) if the target word was semantically unrelated to either of the prime’s two meanings. For unambiguous words, “F” (related) if the target word was semantically related to the prime, and “J” (unrelated) if not. In order to ensure participants successfully realized the semantic relationship between the prime and the target, instructions were presented accompanied by 20 practice trials (not the experimental items) prior to the formal experiment. A trial started with a fixation cross presented for 250 ms in the center of the screen. Then the prime word (i.e., the learned English pseudoword) was presented for 200 ms, followed by a blank screen lasting for 50–100 ms, jittered to decrease anticipation of the onset of the target (Chen et al., 2014). Then the target (i.e., a Chinese word) appeared on the screen until a response was registered or after 3000 ms elapsed with no response. Participants were asked to press “F” (related) or “J” (unrelated) on the keyboard to indicate the relatedness between the prime and the target. The inter-trial interval was 200 ms. In the semantic relatedness judgment task, there were 45 experimental items (one meaning for each of 15 unambiguous words, and two meanings for each of 15 ambiguous words), each paired with a semantically related Chinese word, and a semantically unrelated Chinese word, constituting 90 trials in a block. Each of the 90 pairs was repeated a total of five times, with an inter-repetition trial of at least 11 trials. Note that no more than three trials of the same experimental condition were presented in succession. A short break was granted after every two blocks. In sum, participants completed a total of five blocks, with 90 trials each. The semantic relatedness judgment task took about 40 min.

## Results

### Results of Stroop Task

The accuracy was greater than 85% for all conditions, so it was not analyzed further. In order to validate the Stroop effect, a paired-sample *t*-test was conducted between the incongruent condition and the neutral condition. Results showed the difference reached significance, *t*(43) = 10.51, *p* < 0.001, Cohen’s *d* = 1.583, such that the mean response latency for the incongruent trials (Mean = 796 ms, *SD* = 88) was 87 ms larger than that of the neutral trials (Mean = 708 ms, *SD* = 71). Thus, the Stroop effect was properly captured by the task.

### Results of Study-phase Tests

Given that the one of the major interests of this study was to investigate whether individual differences in inhibitory control ability may contribute to ambiguous word learning, potential factors which may confound with the influence of inhibition control on learning outcomes should be controlled, e.g., the confounding effects of word proficiency. Therefore, in order to ensure the proficiency of unambiguous words did not differ from that of ambiguous words, and that the first and the second meanings were comparable in proficiency, an English-to-Chinese translation production test was administered at the end of each learning day. Then several statistical tests were conducted.

For the translation production results on the first day, a paired-sample t-test was conducted for the production accuracy between unambiguous words (unA) and the dominant meaning of ambiguous words (A-1). Results revealed no significance difference, t(43) = 1.289, p = 0.204, Cohen’s d = 0.194. For the translation production results on the second day, the beginning of the learning of subordinate meaning of ambiguous words (A-2), a repeated measure one-way ANOVA was conducted for the production accuracy with word type (unA, A-1, A-2) as a factor. Results showed no significant differences, *F*(2,86) = 0.631, *p* = 0.534, η^2^ = 0.014. Similarly, a repeated measure one-way ANOVA revealed no significant difference among three conditions on the third day’s data and the fourth day’s data (oral translation production), *F*(2,86) = 0.196, *p* = 0.822, η^2^ = 0.005, *F*(2,86) = 1.000, *p* = 0.372, η^2^ = 0.023, respectively. Results are summarized in **Table 4**. These results suggest that participants had a good master of both unambiguous words and ambiguous words, and that in terms of each meaning, participants’ proficiency for all three groups of pseudoword-meaning pairs did not differ from one another.

**Table 4:** Accuracy (%) of study-phase tests, mean (SD).

Word type	Day 1	Day 2	Day 3	Day 4
unA	97.88 (4.93)	99.09 (2.73)	99.70 (1.41)	99.85 (1.01)
A-1	96.97 (5.83)	99.55 (1.70)	99.70 (1.41)	99.85 (1.01)
A-2	NA	99.24 (2.14)	99.55 (1.70)	99.85 (1.01)


*unA, unambiguous words; A-1, first meaning of ambiguous words; A-2, second meaning of ambiguous words.*

### Results of Semantic Relatedness Judgment Task-Semantic Relatedness Effect

Since the overall accuracy of 90% indicated participants had a good performance in the task, analyses were conducted on response latencies of correct trials only. Extreme outliers (RT < 200 ms or >2500 ms) and response latencies beyond M ± 3SD were excluded (13.4%) from the dataset, retaining 17133 observations. Data were analyzed in the R computing environment (Team, 2013) using linear mixed models (lme4 package, version 0.999999-2; in the R Project for Statistical Computing environment, version 3.0.2) (Baayen et al., 2008; Bates et al., 2014). One important reason for using LME over traditional statistics is that it allows investigation of continuous variables that are based on subject-related differences and item-related differences, which cannot be easily accomplished by traditional ANOVA (Baayen et al., 2008). The LME model was fitted to RT data, with relatedness (related and unrelated) and word type (unA, A-1, A-2) and their interaction as fixed effects, with random intercepts for participants and experimental items and random by-item slopes for relatedness. Then this full model was compared to models with (i) the fixed effect of relatedness removed (ii) the fixed effect of word type removed and (iii) the interaction removed.

Model comparison revealed a significant interaction, χ^2^ = 8.89, df = 2, *p* = 0.01. And for relatedness, the result was χ^2^ = 0, *p* = 1, and for word type, the result was χ^2^ = 0, *p* = 1. Breaking the interaction down, relatedness reached significant for all three word types, for unA, χ^2^ = 10.92, df = 1, *p* < 0.001, for A-1, χ^2^ = 22.75, df = 1, *p* < 0.001, and for A-2, χ^2^ = 4.04, df = 1, *p* = 0.04, indicating semantic representations had been established for all three types of pseudoword-meaning pairs (see **Table 5**).

**Table 5:** Mean response latencies (ms) (SD) for the semantic relatedness judgment task, by relatedness condition and word type.

Semantic relatedness	unA	A-1	A-2
Related	773 (365)	844 (376)	995 (448)
Unrelated	909 (457)	1091 (537)	1090 (546)


*unA, unambiguous words; A-1, first meaning of ambiguous words; A-2, second meaning of ambiguous words.*

### Does Inhibitory Control Ability Modulate Ambiguous Word Learning

In terms of response latency, only the semantically related trials with correct responses were analyzed. Extreme outliers (RT < 200 ms or >2500 ms) and response latencies beyond M ± 3SD were excluded (14.9%) from the dataset, retaining 8418 observations. Stroop effects were transformed to standard scores in order to unify scaling. Overall accuracy was 90%, indicating that participants performed the semantic relatedness judgment task well, so it was not further analyzed. We fit an LME model that included word type, Stroop effect (continuous), and their interaction as fixed effects to predict response latency, with random intercepts for participants and experimental items and random by-item slopes for Stroop effect. We then compared this full model to models with (i) the fixed effect of word type removed (ii) the fixed effect of Stroop effect removed and (iii) the interaction removed.

**FIGURE 3:**
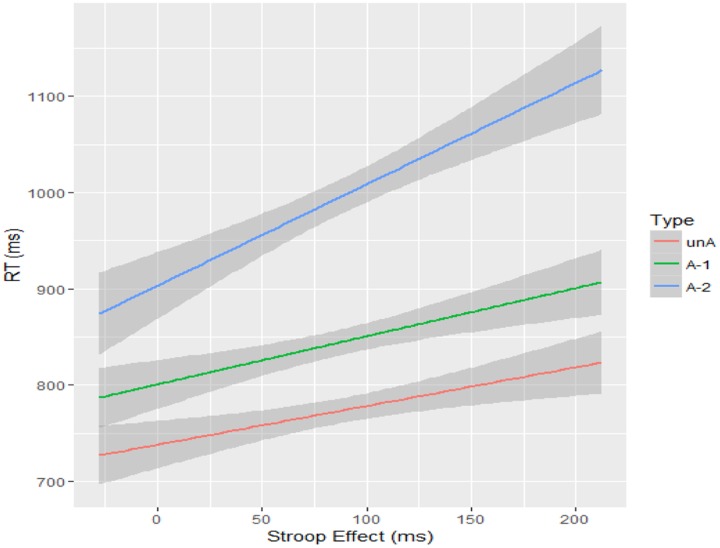
**Response latencies (ms) as the function of word type and inhibition control ability.** Error bands show 95% confidence intervals.

Model comparisons showed an effect of word type (χ^2^ = 26.85, df = 2, *p* < 0.001), such that responses for A-2 were significantly slower than for unA, *z* = 5.73, *p* < 0.001, and A-1, *z* = 4.03, *p* < 0.001. Moreover, there was no significant difference between unA and A-1, *z* = 1.71, *p* = 0.10. The result for inhibition control ability was χ^2^ = 0, *p* = 1. Model comparisons showed an effect of interaction (χ^2^ = 9.16, df = 2, *p* = 0.01). Breaking the interaction down, there was a marginally significant effect for inhibition control ability in A-2 condition, χ^2^ = 3.74, df = 1, *p* = 0.05, suggesting better inhibition control ability indicates faster response in the A-2 condition. However, the effect of inhibitory control ability was not significant either in the unA condition, χ^2^ = 1.60, df = 1, *p* = 0.21, or in the A-1 condition, χ^2^ = 2.10, df = 1, *p* = 0.15, indicating that inhibitory control ability may not be involved in the processing of unambiguous words or the dominant meaning of ambiguous words. **Figure 3** illustrates the response latencies as the function of word type and inhibition control ability.

## Discussion

In the present study, we aimed to investigate what the relationship between representations of new and the prior meanings of newly learned L2 vocabulary words would be, as well as the function of inhibitory control ability on L2 ambiguous word learning. Our results suggested that semantic representations of unambiguous words and ambiguous words were successfully established in the mental lexicon, as was shown by the semantic relatedness effects, i.e., significant difference between the semantically related versus unrelated conditions for all three word types. Moreover, the semantic representation of the second meaning of ambiguous words was weak and unstable compared to the first meaning, as was shown by the significantly slower response latencies for the second meaning. More importantly, the instability of the representation of the second meaning may be partly caused by interference from the prior meaning. Thus inhibitory control ability may modulate the learning of the new meaning, such that individuals with better inhibitory control may more effectively suppress interference from the first meaning and learn the new meanings more quickly. This was shown by the significant predictive ability of inhibition control ability on the performance of the second meaning.

Extending previous monosemous L2 word learning research, our results reveal for the first time that two meanings of ambiguous words differ in the strength of semantic representation at the very early stage of acquisition. One possible mechanism is that the weakness and instability of the representation of the second meaning may stem from the interference cast by semantic representation of the first meaning, and thus the difficulty can be alleviated by better inhibitory control. We postulate that the obvious disadvantage of the second meaning may result from the difficulty when integrating into one-to-many form-meaning connections. In particular, when participants start to learn the second meaning of the ambiguous words, the first meaning learned 1 day earlier has already established one-to-one mapping, which needs to be revised when the new meaning is encountered. It is probably the interference induced by the first meaning that creates difficulty in learning the new meaning. Hence, learners will have weaker representation of the new meaning.

However, since in the present study the new meaning was presented just 1 day after the first meaning, and the learning tasks for both meanings lasted until the testing phase, there is a possibility that the difficulty of second meaning was heightened by a comparatively shorter exposure than for the first meaning. However, we tried to minimize this possibility. Firstly, during the learning phase, instead of presenting each meaning for a fixed number of times, participants used a flexible self-paced learning mode, in which learners could review items as many times as possible. Therefore, the decrement of the less-frequent exposure would be lessened to large extent. Secondly, the learning outcome during the learning phase was recorded everyday using an L2-to-L1 translation production task. Results showed no significant differences among the three types of pseudoword-meaning pairs for any of the training/testing days. This finding suggests that learners could memorize and retrieve the second meaning as well as the first meaning. Furthermore, the study conducted by Degani et al. (2014) revealed that order of acquisition effects in L1 ambiguous word learning could be minimized by controlling for frequency effects, by presenting the first translation for six times in session one and the second translation for six times in session two interleaved with a 2-day break (Degani et al., 2014). They posited that is was the order of acquisition rather than exposure that led to the difficulty in learning the second translation. They also examined whether teaching the two meanings simultaneously could alleviate the translation-ambiguity learning disadvantage. Results showed higher accuracy in the together training condition than the separate training condition. They posited that presenting two meanings together may aid participants in assigning the appropriate associative strength to each of the two lexical links, and in so doing prevented them from needing to revise their initial association structure. Nevertheless, in real-life L2 vocabulary learning, simultaneous learning is rarely the case. As an example, to teach a Chinese learner of English the meaning of the word “run,” which has more than 30 senses in the dictionary, it may be better to teach high frequency and high concreteness senses early on. Then, with the improvement of learners’ English proficiency, cognitive skills, and life experience, extend the learning of “run” to more abstract meanings. Learning different word senses separately, as in Degani et al. (2014), is ecologically valid. Most learners are not exposed to all possible senses of polysemous words, but once learned, several senses can be compared and contrasted with one another to help learners gain awareness of the general and distinctive features of the senses. Therefore, in the current study, for the reasons addressed above, the two meanings of the ambiguous words were presented separately with an interval of 1 day, and when participants started to learn the second meaning, the first meaning was presented simultaneously on the flashcard, aiding in the establishment of one-to-many mappings. Results showed that the performance of the second meaning was correlated with inhibitory control ability. Therefore, the beneficial role of inhibition in ambiguous word learning is well-grounded, such that in the establishment of the semantic representation of the new meaning, individuals with better inhibitory control may more efficiently suppress interference brought by the primary meaning, and thus establish stable representations of the new meanings more quickly.

Another interesting finding of our study is that despite the competing relationship between the previously learned and later-learned meanings, the interference seems to be unidirectional. That is, the first meaning may interfere with the establishment of the second meaning, but not vice versa, as shown by the predictive ability of inhibitory control in only the second meaning condition. These findings shed light upon our understanding of sense creating mechanisms. Our findings showed that at the very early stage of learning, meaning creation may be refined to a one-way inhibitory mechanism. In fact, lexical and semantic representations, whether in a native language or a second language, are dynamic and ever-changing from the moment of their formation. In that sense, the way that a new sense is created may impact the way it will be represented in the semantic space. To study ambiguous words from the perspective of how a new sense is added to lexical representations and what the consequent relations between the first sense and the new sense would be is crucial to understanding lexical ambiguity relationships and their development in L2 lexical networks. Meaning creation and integration for ambiguous words require change of the weights assigned to each of the form-meaning associations. There are logical reasons to assume that when a new meaning is presented, the integration process from one-to-one form-meaning mapping to one-to-many form-meaning mappings is supposed to negatively affect both meanings. And inhibitory control ability is supposed to correlate with both meanings. However, as our results showed, inhibitory control only predicted performance on the second meaning, suggesting that inhibitory connections are unidirectional. Notably, there was no significant difference in response latencies between the first meaning and the unambiguous word conditions. This may point to an ordered accessing mechanism. In particular, when participants saw the target and needed to decide if it was semantically related to either of the learned meanings, access to the first meaning was prioritized. If the target was related to the first meaning, decision-making was complete. If the target was not related to the first meaning, the participant would need to check for additional meanings, which would take more time. This interpretation is consistent with a previous study by Medina et al. (2011), in which learners generated a single conjecture about a word (in our study, the first meaning) and sought its confirmation on later encounters. The unidirectional inhibitory connection at the initial stage of learning may reveal an ordered meaning creating mechanism. Future studies may take these findings a step further by examining when and how the later-learned and usually non-dominant meanings would start to affect the initial meaning.

With respect to lexical ambiguity, there are different kinds of semantic ambiguity in traditional linguistic research. Recent research has emphasized the importance of the degree of semantic relatedness between the meanings of a polysemous word in explaining ambiguity effects (Rodd et al., 2002; Klepousniotou and Baum, 2007; Armstrong and Plaut, 2008). Thus, it may be important to consider the degree of meaning relatedness of the ambiguous words. Since the present study was confined to ambiguous words with semantically unrelated meanings, generalizations should be made cautiously concerning semantically related senses. Future studies may extend the present study in investigating the influence of sense relatedness on ambiguous word learning.

## Conclusion

To our knowledge, this is the first study to examine the relationship of multiple semantic representations in learning of L2 ambiguous words, as well as examining how inhibitory control functions during L2 ambiguous word learning. The findings suggest that the representation of the second meaning of L2 ambiguous word is weak at the initial stage of learning, partly due to interference by the representation of the previously learned meaning. Moreover, inhibitory control ability may modulate learning of the new meaning, such that individuals with better inhibitory control may more effectively suppress interference from the first meaning, and thus learn the new meaning more quickly.

## Author Contributions

YL and BC designed the experiment and wrote the manuscript; YL collected and performed data analysis; JW performed data analysis, BC and SD edited and revised the manuscript.

## Conflict of Interest Statement

The authors declare that the research was conducted in the absence of any commercial or financial relationships that could be construed as a potential conflict of interest.

## Footnotes

1 http://crr.ugent.be/programs-data/wuggy
