# Supplementary material for: The Inhibitory Mechanism in Learning Ambiguous Words in a Second Language
Source: Front Psychol. 2017 Apr 27;8:636. doi: 10.3389/fpsyg.2017.00636 (PMC5406400; doi:10.3389/fpsyg.2017.00636)
Supplement: Supplementary file 1 [file Table_1.DOCX]

| **Appendix A. Learning items** | | | | | |  |
| --- | --- | --- | --- | --- | --- | --- |
| **Unambiguous Words** | |  | **Ambiguous Words** | | | |
| **Pseudoword** | **Meaning** |  | **Pseudoword** | **1^st^ Meaning** | **2^nd^ Meaning** | |
| sessand | 水稻(rice) |  | mectoun | 椅子(chair) | 玉米(corn) | |
| rackarn | 床铺(bed) |  | mossack | 手掌(palm) | 汽油(gasoline) | |
| chefawn | 帽子(hat) |  | ranctay | 袖子(sleeve) | 胡须(beard) | |
| kursten | 种子(seed) |  | foctoun | 厨房(kitchen) | 司机(driver) | |
| pregild | 相机(camera) |  | rephern | 小溪(brook) | 电视(television) | |
| cirweat | 苹果(apple) |  | copfome | 羽毛(feather) | 士兵(soldier) | |
| stospeme | 电灯(lamp) |  | jimpsen | 秋天(autumn) | 头颅(skull) | |
| nawdew | 夜晚(night) |  | fiqutz | 橘子(orange) | 公路(highway) | |
| pregith | 血管(blood vessel) |  | stospelt | 轮船(ship) | 校长(headmaster) | |
| provuth | 腹部(stomach) |  | soltoor | 商店(store) | 鼻子(nose) | |
| rartcay | 泥土(dirt) |  | clactean | 沙发(sofa) | 蝴蝶(butterfly) | |
| fludpet | 耳朵(ear) |  | tiqoud | 火柴(matchstick) | 麻雀(sparrow) | |
| spactien | 日照(sunshine) |  | conswist | 水泥(cement) | 狮子(lion) | |
| elpeme | 骆驼(camel) |  | gonipt | 毛孔(pore) | 公园(park) | |
| fatause | 舌头(tongue) |  | lifuch | 口腔(oral cavity) | 棉花(cotton) | |
| Note: The English translations of the original Chinese materials are presented in parenthesis. | | | | | | |

| **Appendix B. Stimuli used in semantic relatedness judgment task—Unambiguous words.** | | | |
| --- | --- | --- | --- |
| **Pseudoword Prime** | **Meaning** | **Related Target** | **Unrelated Target** |
| sessand | 水稻(paddy) | 大米(rice) | 肥皂(soap) |
| rackarn | 床铺(bed) | 睡衣(pajama) | 金鱼(goldfish) |
| chefawn | 帽子(hat) | 头部(head) | 白菜(cabbage) |
| kursten | 种子(seed) | 秧苗(seedling) | 杂志(magazine) |
| pregild | 相机(camera) | 照片(photo) | 果汁(juice) |
| cirweat | 苹果(apple) | 桃子(pea) | 镜子(mirror) |
| stospeme | 电灯(lamp) | 光线(light) | 糖果(candy) |
| nawdew | 夜晚(night) | 月亮(moon) | 棋子(chess pieces) |
| pregith | 血管(blood vessel) | 静脉(vein) | 盘子(plate) |
| provuth | 腹部(stomach) | 肚子(belly) | 桃花(peach flower) |
| rartcay | 泥土(dirt) | 灰尘(dust) | 钢琴(piano) |
| fludpet | 耳朵(ear) | 声音(sound) | 面粉(flour) |
| spactien | 日照(sunlight) | 阳光(sunshine) | 刀子(knife) |
| elpeme | 骆驼(camel) | 沙漠(desert) | 头发(hair) |
| fatause | 舌头(tongue) | 嘴巴(mouth) | 学校(school) |
| Note: the Meaning column did not appear in the test, neither did the English translations presented in parentheses. | | | |

| **Appendix C. Stimuli used in semantic relatedness judgment task—Ambiguous words.** | | | |  |
| --- | --- | --- | --- | --- |
| **Pseudoword**  **prime** | **1^st^ Meaning**  **2^nd^ Meaning** | **Related Targets** | **Unrelated Targets** | |
| mectoun | 椅子(chair) | 板凳(stool) | 零钱(change) | |
|  | 玉米(corn) | 谷物(cereal) | 围巾(scarf) | |
| mossack | 手掌(palm) | 指头(finger) | 火车(train) | |
|  | 汽油(gasoline) | 燃料(fuel) | 护士(nurse) | |
| ranctay | 袖子(sleeve) | 衣服(clothes) | 西瓜(watermelon) | |
|  | 胡须(beard) | 男人(male) | 绿豆(mung bean) | |
| foctoun | 厨房(kitchen) | 炊烟(smoke from kitchen chimneys) | 雷达(radar) | |
|  | 司机(driver) | 汽车(vehicle) | 粉笔(chalk) | |
| rephern | 小溪(brook) | 河流(river) | 筷子(chopsticks) | |
|  | 电视(television) | 屏幕(screen) | 蚂蚁(ant) | |
| copfome | 羽毛(feather) | 翅膀(wing) | 袜子(socks) | |
|  | 士兵(soldier) | 军人(serviceman) | 太阳(sun) | |
| jimpsen | 秋天(autumn) | 季节(season) | 孩子(kid) | |
|  | 头颅(skull) | 脑袋(cranium) | 地板(floor) | |
| fiqutz | 橘子(orange) | 柑橘(citrus) | 电池(battery) | |
|  | 公路(highway) | 高速(expressway) | 青蛙(frog) | |
| stospelt | 轮船(ship) | 甲板(deck) | 肝脏(liver) | |
|  | 校长(headmaster) | 老师(teacher) | 农场(farm) | |
| soltoor | 商店(store) | 百货(grocery) | 眉毛(eyebrow) | |
|  | 鼻子(nose) | 空气(air) | 手枪(pistol) | |
| clactean | 沙发(sofa) | 躺椅(lounge) | 水藻(algae) | |
|  | 蝴蝶(butterfly) | 蜜蜂(bee) | 杯子(cup) | |
| tiqoud | 火柴(matchstick) | 蜡烛(candle) | 绵羊(sheep) | |
|  | 麻雀(sparrow) | 乌鸦(crow) | 毛衣(sweater) | |
| conswist | 水泥(cement) | 沙子(sand) | 橡皮(eraser) | |
|  | 狮子(lion) | 老虎(tiger) | 书包(backpack) | |
| gonipt | 毛孔(pore) | 皮肤(skin) | 房子(house) | |
|  | 公园(park) | 广场(plaza) | 匕首(dagger) | |
| lifuch | 口腔(oral cavity) | 牙齿(tooth) | 油漆(oil paint) | |
|  | 棉花(cotton) | 被子(quilt) | 咖啡(coffee) | |
| Note: the Meaning column did not appear in the test, neither did the English translations presented in parentheses. | | | |  |
